# Identifying the origin of socioeconomic disparities in outcomes of major elective operations^☆^

**DOI:** 10.1016/j.sopen.2023.04.001

**Published:** 2023-04-15

**Authors:** Catherine G. Williamson, Shannon Richardson, Shayan Ebrahimian, Elsa Kronen, Arjun Verma, Peyman Benharash

**Affiliations:** Cardiovascular Outcomes Research Laboratories (CORELAB), David Geffen School of Medicine at UCLA, Los Angeles, CA, United States of America

**Keywords:** Socioeconomic status, Surgical outcomes, Disparities, Nationwide readmissions database

## Abstract

**Background:**

While the impact of socioeconomic status (SES) on surgical outcomes has been examined in limited series, it remains a significant determinant of healthcare outcomes at the national level. Therefore, the current study aims to determine SES disparities at three time-points: hospital accessibility, in-hospital outcomes, and post-discharge consequences.

**Methods:**

The Nationwide Readmissions Database 2010–2018 was used to isolate major elective operations. SES was assigned using previously coded median income quartiles as defined by patient zip-code, with *low SES* defined as the lowest quartile and *high SES* as the highest.

**Results:**

Of an estimated 4,816,837 patients undergoing major elective operations, 1,037,689 (21.3 %) were categorized as *low SES* and 1,288,618 (26.5 %) as *high*. On univariate analysis and compared to those of *low SES, high SES* patients were more frequently treated at high-volume centers (70.9 vs 55.6 %, p < 0.001), had lower rates of in-hospital complications (24.0 vs 29.0 %, p < 0.001) and mortality (0.4 vs 0.9 %, p < 0.001) as well as less frequent urgent readmissions at 30- (5.7 vs 7.1 %, p < 0.001) and 90-day timepoints (9.4 vs 10.7 %, p < 0.001). On multivariable analysis, *high SES* patients had higher odds of treatment at high-volume centers (Odds: 1.87, 95 % CI: 1.71–2.06), and lower odds of perioperative complications (Odds: 0.98, 95 % CI: 0.96–0.99), mortality (Odds: 0.70, 95 % CI: 0.65–0.75), and urgent readmissions at 90-days (Odds: 0.95, 95 % CI: 0.92–0.98).

**Conclusion:**

This study fills a much-needed gap in the current literature by establishing that all of the aforementioned timepoints include significant disadvantages for those of low socioeconomic status. Therefore, a multidisciplinary approach may be required for intervention to improve equity for surgical patients.

## Introduction

Socioeconomic status (SES) disparities in surgical outcomes have been widely documented [[1], [2], [3]]. This relationship has been described across several major operations including but not limited to oncologic, cardiovascular, and orthopedic procedures [[4], [5], [6], [7], [8]]. Despite robust documentation of these inequities, limited understanding of the underlying mechanisms contributing to these disparities has hindered development of effective interventions. While these mechanisms are complex and multifaceted, many have posited explanations. Several studies site SES to be associated with disease-modifying and health-seeking behavior [2,9,10]. Others have reported quality being attributable to where people tend to receive care [5,11]; disparities are evident in not only timing of surgical management [12] but also in access to quality surgical care [13].

In addition to these factors, several series have noted disparities in readmission rates. For example, a study assessing national Medicare claims data found Black patients had higher odds of readmission following major surgery with White as reference [6]. Similarly, Medberry and colleagues reported socioeconomic factors were associated with lobectomy readmission for patients with early stage lung cancer [4]. While many have documented SES disparities in surgical outcomes, there is lack of clarity on how to best intervene to mitigate these gaps. Thus, understanding which timepoint—pre-, inter-, or post-hospitalization—contributes to such disparities is critical for improvement.

The current retrospective cohort study aims to determine SES disparities at three time-points: 1) hospital accessibility, 2) in-hospital outcomes and 3) post-discharge consequences. We hypothesized SES to be independently associated with reduced hospital access as well as inferior in-hospital and post-discharge outcomes.

## Methods

The Nationwide Readmissions Database (NRD) from 2010 to 2018 was utilized to identify all elective adult noncardiac operations. The NRD is the largest publicly available all-payer readmissions database in the U.S. and provides accurate estimates for 58 % of annual hospitalizations using survey weighted methodology. The operations included pneumonectomy, colectomy, hepatectomy, abdominal aortic aneurysm (AAA) repair, pancreatectomy, gastrectomy, and esophagectomy, as has been described [14,15]. Relevant patient, operative and hospital characteristics were extracted using the *International Classification of Diseases, Ninth and Tenth Revisions* (ICD-9/10) codes and the Healthcare Cost and Utilization Project data dictionary. Variables such as insurance status, income, and sex were defined according to the NRD data dictionary. SES was assigned using previously coded median income quartiles as defined by patient zip-code, with *low SES* defined as the lowest quartile and *high SES* as the highest. Patients with missing values for mortality, income, or insurance were excluded from analysis (2.6 %). Centers were classified as low-, medium- or high-volume based on the annual case-volume of operations with cut-offs at the 33rd and 66th percentiles. The Elixhauser Comorbidity Index, a previously validated composite of 30 chronic conditions, was used to quantify the burden of comorbidities for each patient [16]. Further, hospitalization costs were calculated using center-specific cost-to-charge ratios and were inflation adjust to the 2018 Personal Healthcare–Hospital Index [17].

Categorical and continuous variables are reported as frequency (%) and means with standard deviation (SD). Pearson chi-square and adjusted Wald tests were used to compare categorical and continuous variables, respectively. The primary outcome of interest was to assess the independent association of SES with patient outcomes at the three predetermined timepoints and assess for differences based on SES. To evaluate this relationship, multivariable models were generated with Elastic Net methodology [18]. This approach provides penalized selection of variables to maximize out-of-sample validity and reduce the potential for overfitting. The models were tested using 10-fold cross-validation [19]. Variables ultimately chosen for the model included patient age, sex, income, insurance, indication for operation, operation type, Elixhauser comorbidity index, hospital teaching status, hospital volume, and calendar year. Outcomes are reports as adjusted odds ratio (AOR) and beta coefficients, as appropriate, with 95 % confidence intervals (95 % CI). An α < 0.05 was considered statistically significant. All statistical analyses were performed using Stata 16.1 [20]. This study was deemed exempt from full review by the Institutional Review Board at the University of California, Los Angeles.

## Results

Of an estimated 4,816,837 patients undergoing major elective operations, 1,037,689 (21.3 %) were categorized as *low SES* and 1,288,618 (26.5 %) as *high*. *Low SES* were on average younger (63.7 vs 64.5 years, p < 0.001), more frequently female (54.8 vs 53.7 %, p < 0.001), and had higher burden of comorbidities as defined by the Elixhauser Index (2.51 vs 2.11 points, p < 0.001, Table 1). Further, these patients had lower rates of private insurance (31.1 vs 46.0 %, p < 0.001) and higher rates of all comorbidities including coronary artery disease (13.0 vs 10.6 %, p < 0.001), cancer (24.7 vs 20.1 %, p < 0.001), diabetes (19.4 vs 12.9 %, p < 0.001), hypertension (59.8 vs 52.1 %, p < 0.001), and renal failure (6.2 vs 4.9 %, p < 0.001, Table 1). Compared to those of highest income quartile, *low SES* patients were less frequently treated at high-volume centers (55.6 vs 70.9 %, p < 0.001) or centers in large metropolitan areas (39.6 vs 81.8 %, p < 0.001). *Low SES* patients more frequently underwent pneumonectomy (4.3 vs 3.8 %, p < 0.001), gastrectomy (3.2 vs 2.5 %, p < 0.001), and colectomy (24.5 vs 17.4 %, p < 0.001, Table 1).

**Table 1:** Univariate clinical, demographic and hospital factors by income level in percentile (SD: standard deviation, AAA: abdominal aortic aneurysm).

	0-25th	26-50th	51-75th	76-100th	p-Value
Demographics					
Age (years, SD)	63.7 (12.3)	64.5 (12.2)	64.4 (12.5)	64.5 (12.7)	<0.001
Female (%)	54.8	54.5	54.3	53.7	<0.001
Elixhauser Comorbidity Index (points, SD)	2.5 (1.8)	2.4 (1.8)	2.3 (1.8)	2.1 (1.8)	<0.001
Insurance (%)					<0.001
Medicare	55.6	54.9	52.0	48.8	
Medicaid	8.6	5.6	4.1	2.6	
Private	31.2	35.9	40.6	46.0	
Uninsured	1.4	0.9	0.7	0.5	
Comorbidities (%)					
Alcohol abuse	2.2	1.9	1.8	1.6	<0.001
Arrythmias	13.2	13.3	13.0	12.9	<0.001
Drug abuse	1.6	1.3	1.2	1.0	<0.001
Coronary artery disease	13.0	12.7	11.7	10.6	<0.001
Cancer	24.7	22.2	20.7	20.1	<0.001
Congestive heart failure	4.6	4.1	3.6	3.0	<0.001
Coagulopathy	2.6	2.5	2.4	2.6	<0.001
Chronic obstructive pulmonary disease	2.1	1.9	1.7	1.5	<0.001
Diabetes mellitus	19.4	17.3	15.6	12.9	<0.001
Hypertension	59.8	57.1	55.3	52.1	<0.001
Obesity	17.3	17.6	17.5	16.1	<0.001
Liver disease	2.8	2.6	2.5	2.5	<0.001
Renal failure	6.2	5.8	5.6	4.9	<0.001
Tobacco use	14.9	12.1	10.2	7.2	<0.001
Hospital factors (%)					
High-volume center	55.6	58.0	62.9	70.9	<0.001
Hospital urban-rural designation					<0.001
Large metropolitan	39.6	40.8	55.8	81.8	
Small metropolitan	46.2	47.2	39.8	17.7	
Micropolitan	11.6	9.7	3.6	0.5	
Teaching center	60.9	60.1	66.2	73.2	<0.001
Operation (%)					
Hepatectomy	2.4	2.2	2.2	2.5	<0.001
Pneumonectomy	4.3	4.2	4.0	3.8	<0.001
Gastrectomy	3.2	2.8	2.7	2.5	<0.001
Colectomy	24.5	21.8	20.1	17.4	<0.001
Esophagectomy	0.9	0.9	0.9	0.8	<0.001
AAA repair	1.0	0.8	0.8	0.6	<0.001
Pancreatectomy	3.0	2.9	2.9	3.1	<0.001

On univariate analysis, *low SES* patients experienced higher rates of mortality (0.9 vs 0.4 %, p < 0.001), complications (29.0 vs 24.0 %, p < 0.001), nonhome discharge (46.5 vs 40.4 %, p < 0.001), 30-day readmissions (7.0 vs 5.6 %, p < 0.001), and 90-day readmissions (10.6 vs 8.4 %, p < 0.001, Table 2). Indications for readmissions are shown in Table 3. Patients in the lowest income quartile were readmitted for infectious (12.8 vs 12.0 %, p < 0.001) and respiratory indications (6.9 vs 5.4 %, p < 0.001, Table 3) more commonly than those of *high SES*. Notably, costs for *high SES* patients were on average greater ($22,217 vs 20,888, p < 0.001) than those within the *low SES* group (Table 2).

**Table 2:** Univariate outcomes by income level in percentile (SD: standard deviation).

Outcomes	0-25th	26-50th	51-75th	76-100th	p value
Mortality (%)	0.9	0.7	0.6	0.4	<0.001
Non-home discharge (%)	46.5	47.9	46.3	40.4	<0.001
Cost ($1000, SD)	20.9 (20.7)	21.0 (19.8)	20.9 (20.3)	22.2 (22.1)	<0.001
Hospital duration (days, SD)	5.2 (6.4)	4.7 (5.9)	4.4 (5.9)	4.2 (5.6)	<0.001
30-day readmission (%)	7.0	6.3	6.1	5.6	<0.001
90-day readmission (%)	10.6	9.6	9.1	8.4	<0.001
Complications (%)	29.0	27.1	25.5	24.0	<0.001

**Table 3:** Univariate primary readmission indications by income level in percentile.

Readmission indication (%)	0-25th	26-50th	51-75th	76-100th	p value
Vascular	1.5	1.5	1.4	1.3	<0.001
Hepatobiliary	2.6	2.4	2.3	2.6	<0.001
Infectious	12.8	12.1	12.1	12.0	<0.001
Endocrine	2.4	2.3	2.1	2.1	<0.001
Renal	1.9	1.7	1.6	1.3	<0.001
Gastrointestinal	19.9	19.7	19.9	19.4	0.12
Respiratory	6.9	6.3	5.7	5.4	<0.001
Cardiovascular	9.0	9.1	9.1	8.8	0.38
Hematologic	1.7	1.5	1.5	1.6	<0.001
Neurologic	1.9	1.9	1.9	1.9	0.65
Psychiatric	1.1	0.9	0.8	0.8	<0.001

**Fig. 1:**
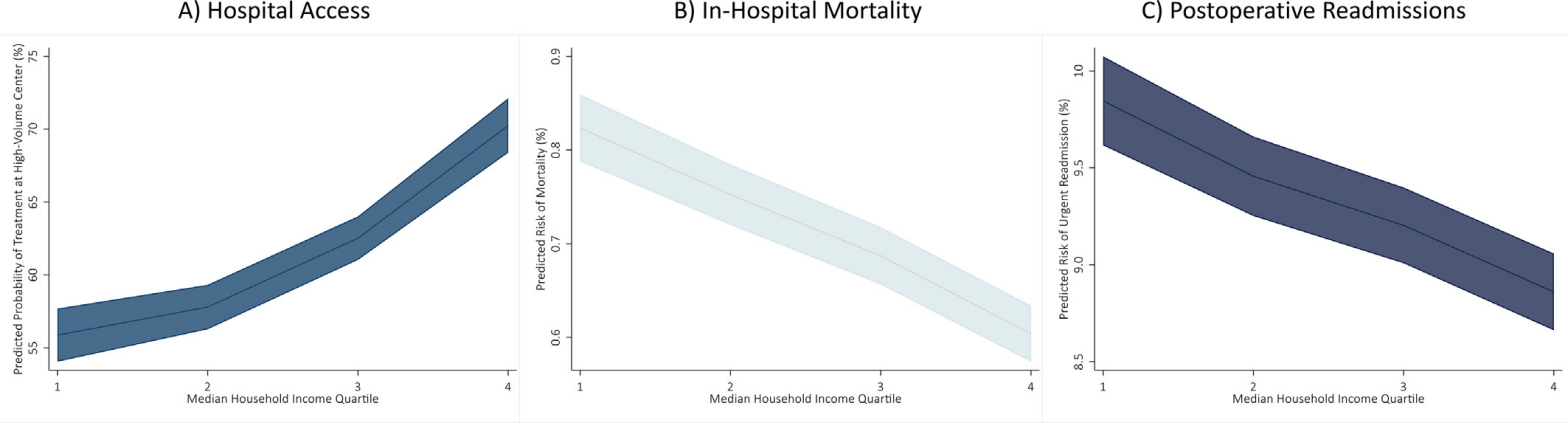
Predicted likelihood of A) high-volume hospital access, B) in-hospital mortality, and C) postoperative readmissions by income level in quartile

**Fig. 2:**
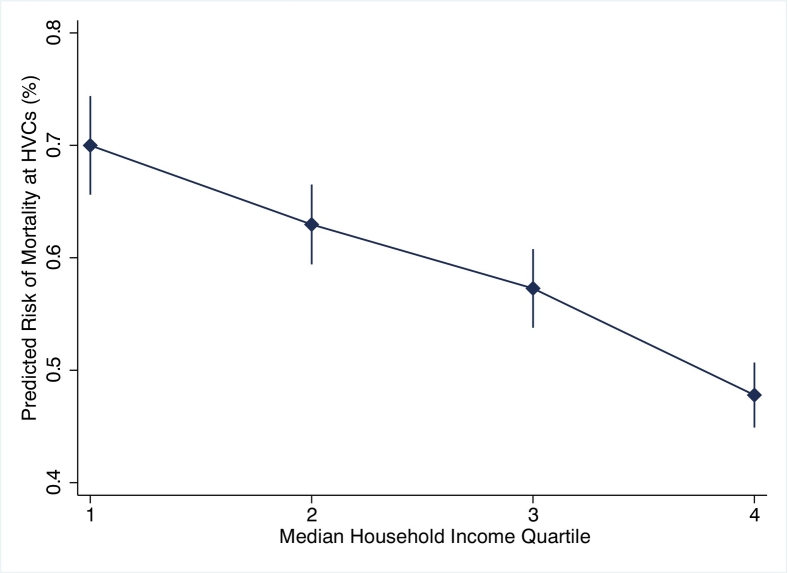
Predicted risk of mortality by income level in quartile for solely high-volume centers

**Fig. 3:**
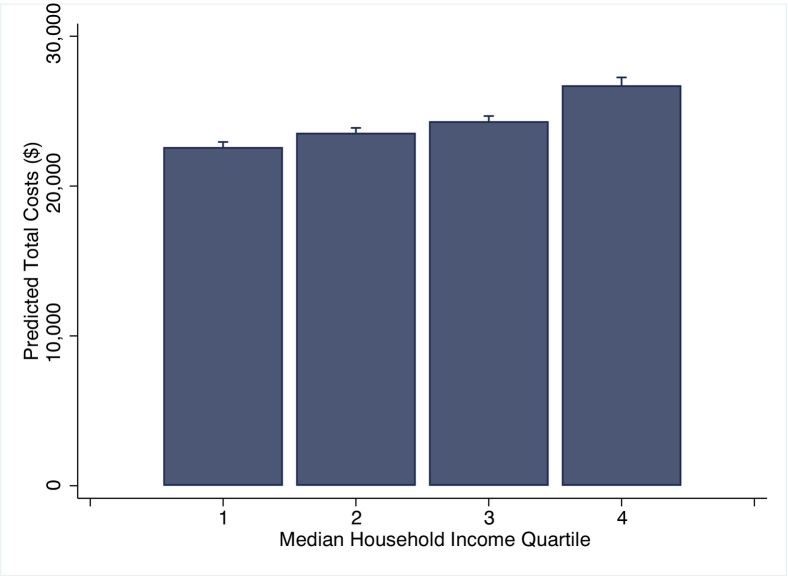
Predicted cumulative costs per patient by income level in quartile

After adjustment for patient and hospital level factors, *high SES* patients had higher odds of treatment at high-volume centers (odds: 1.87, 95 % CI: 1.71–2.06, Fig. 1A), and lower odds of perioperative complications (odds: 0.98, 95 % CI: 0.96–0.99), mortality (odds: 0.70, 95 % CI: 0.65–0.75, Fig. 1B), and urgent readmissions at 90-days (odds: 0.95, 95 % CI: 0.92–0.98, Fig. 1C) with *low SES* as reference. Moreover, in a subgroup analysis examining solely patients at high-volume centers, outcomes remained superior for *high SES* patients (Fig. 2). Total costs of index and readmissions per patient after adjusted analysis are shown in Fig. 3. The *high SES* patients costs more overall ($26,726 vs 22,605, p < 0.001) for the index hospitalization plus any additional readmission hospitalizations (Fig. 3), while having a shorter hospitalization duration (4.2 vs 5.2 days, p < 0.001).

## Discussion

In our study, *low SES* patients had reduced access to high-volume surgical centers compared to *high SES* counterparts. Moreover, this cohort experienced inferior clinic outcomes during the perioperative period, as noted by higher odds of in-hospital mortality and complications. Notably, these suboptimal clinical outcomes were maintained for *low SES* patients even when treated at high-volume surgical centers. Finally, post-operative non-elective readmissions were greater for the *low SES* cohort at both 30- and 90-day intervals. While this study fills a much-needed gap in the current literature by establishing that all of the aforementioned timepoints are critical for intervention in order to improve equity for surgical patients, many of these findings require further discussion.

Social determinants of health have been shown to significantly impact access to surgical care in multiple sectors [21]. Our study noted an almost 2-fold odds of treatment at a high-volume surgical center for *High SES* patients compared to *low SES*. Decreased access to timely operations may be fueled by decreased access to high-quality primary care, prohibitive costs without adequate insurance coverage, late detection, or systemic delays in referral for a surgical consultation [[22], [23], [24], [25], [26]]. In fact, in a study by Martin and colleagues, 80 % of elective operations were offered to those with high incomes within a study cohort of bariatric patients [24]. As our cohort solely included those who received operative management, the disparities are likely even greater than exhibited in this study due to a large proportion of *low SES* patients not referred or deemed ineligible for surgery. Moreover, as the elective surgical population relies heavily on outpatient referral for definitive management, improvements in surgical access require a collaborative effort between primary care providers and surgeons to decrease times to operation. While the current study cannot capture the multifaceted nature of social determinants of health, this study indicates differences exist in surgical access between those with *low SES* versus *high SES*. Further studies are required to investigate strategies to improve this collaboration and, in doing so, improve access to a high-risk and underserved population.

Regardless of treatment at high-volume centers, the present work noted *low SES* patients continued to suffer inferior perioperative outcomes than *high SES* patients. Many have attributed this disparity to variation in clinical severity at the time of presentation [27]. Though differences in case complexity are difficult to assess using an administrative database, our study matched patients based on comorbidities at the time of admission, indicating additional explanations may be plausible. The current study showed *high SES* patients cost more than *low SES* while being hospitalized for a shorter duration. While hospitalization duration and costs are commonly correlated, this study showed a divergence from that trend. A potential cause for this surprising finding is greater resources utilized for the *high SES* cohort, which may incur higher costs while offering superior outcomes to those within higher income groups. In fact, in a global study of cancer operations, patients treated at centers with greater infrastructure experienced improved clinical outcomes compared to those cared for at low resource locations [28]. Regardless of the mechanism underlying these cost disparities, our results point to the necessity for equal treatment for all surgical patients at each center. Moreover, as lower income individuals remain at higher risk for perioperative complications than high-income, increased monitoring and management of this group may lead to improved surgical endpoints.

Postoperative management may require significant social support, scheduled follow-up visits, and complex care coordination. This study found *low SES* patients suffered increased odds of non-elective readmissions at both 30- and 90-day timepoints. Urgent readmissions may indicate a lacking postoperative support system, greater complication occurrence, or deficient follow-up care. In an institutional study of Medicare patients, those living in high-poverty areas were 24 % more likely to be urgently readmitted at 30 days [29]. This effect may be explained by the fact that those living in a poor neighborhood may have more difficulty in accessing post-discharge resources, primary care follow-up, or local support. Another postulation is that patients in lower income categories may utilize emergency departments for their primary care. In fact, in a study by Cheung et al., Medicaid beneficiaries utilized the emergency department 1.5 times as frequently as those privately insured, likely as an access point for nonemergent care [30]. However, adequate post-operative care by the surgical team is critical to the success of the patient and their operation and is not easily substituted by an emergency department with variable access to patient history. Moreover, overuse of emergency department and urgent care visits may further drive healthcare costs which are not captured in the present work. A possible intervention for this population may be increased emphasis on social work postoperatively, which has been shown to ease transitions to home, establish social support, and improve postoperative follow-up [31]. Therefore, it is crucial to implement dedicated post-discharge pathways for post-operative patients, regardless of socioeconomic status.

As an administrative database, the NRD is limited by its reliance on hospital billing preferences and manual coding, which may affect the study data. Moreover, the retrospective nature of this work precludes any causal conclusions. Further, the NRD is unable to provide specific clinical information, including illness severity, lab values, or imaging. The patients within this cohort span a wide range of surgical difficulty and clinical severity, which cannot be fully captured without granular clinical data. Importantly, SES is a challenging factor to quantify simply with median income of the patients' zip code within the study due to its highly complex nature. Specifically, the NRD does not include variables for race or ethnicity, which may affect study outcomes due to the multifactorial nature of socioeconomic status. Despite these limitations, we utilized the largest, all-payer, readmissions database to evaluate the best timepoint for intervention to improve outcomes for *low SES* patients within the surgical pathway.

In conclusion, all three of the timepoints: pre, peri, and postoperative exhibit inferior outcomes for *low SES* patients, likely due to a multitude of factors comprising social determinants of health. However, this study indicates that these time points all require intervention in order to improve equity in outcomes for surgical patients. Improvements must be made through a concerted effort across primary care physicians, surgical teams, and social workers to ensure adequate access, optimal outcomes, and ample follow-up for *low SES* communities undergoing major operations.

## Funding sources

The authors have no financial conflicts of interests to disclose.

## Ethical approval statement

This study was deemed exempt from full review by the Institutional Review Board at the University of California, Los Angeles.

## Credit authorship contribution statement

CW, SR, and SE conceptualized the study, wrote, and reviewed the manuscript and conducted the data collection. EK and AV conducted data collection for the study, read and reviewed the final manuscript. PB conceptualized the study, oversaw all data collection, wrote, and reviewed the final manuscript.

## Conflict of interest

The authors report no proprietary or commercial interest in any product mentioned or concept discussed in this manuscript.
